# Development of a simulation-based curriculum for Pediatric prehospital skills: a mixed-methods needs assessment

**DOI:** 10.1186/s12873-021-00494-4

**Published:** 2021-09-25

**Authors:** Kevin A. Padrez, John Brown, Andy Zanoff, Carol C. Chen, Nicolaus Glomb

**Affiliations:** 1 Department of Emergency Medicine, University of California, San Francisco, USA; 2 San Francisco EMS Agency, San Francisco, USA; 3 San Francisco Fire Department, San Francisco, USA

**Keywords:** Simulation, Pediatrics, Education

## Abstract

**Background:**

The assessment and treatment of pediatric patients in the out-of-hospital environment often presents unique difficulties and stress for EMS practitioners**.**

**Objective:**

Use a mixed-methods approach to assess the current experience of EMS practitioners caring for critically ill and injured children, and the potential role of a simulation-based curriculum to improve pediatric prehospital skills.

**Methods:**

Data were obtained from three sources in a single, urban EMS system: a retrospective review of local pediatric EMS encounters over one year; survey data of EMS practitioners’ comfort with pediatric skills using a 7-point Likert scale; and qualitative data from focus groups with EMS practitioners assessing their experiences with pediatric patients and their preferred training modalities.

**Results:**

2.1% of pediatric prehospital encounters were considered “critical,” the highest acuity level. A total of 136 of approximately 858 prehospital providers responded to the quantitative survey; 34.4% of all respondents either somewhat disagree (16.4%), disagree (10.2%), or strongly disagree (7.8%) with the statement: “I feel comfortable taking care of a critically ill pediatric patient.” Forty-seven providers participated in focus groups that resulted in twelve major themes under three domains. Specific themes included challenges in medication dosing, communication, and airway management. Participants expressed a desire for more repetition and reinforcement of these skills, and they were receptive to the use of high-fidelity simulation as a training modality.

**Conclusions:**

Critically ill pediatric prehospital encounters are rare. Over one third of EMS practitioners expressed a low comfort level in managing critically ill children. High-fidelity simulation may be an effective means to improve the comfort and skills of prehospital providers.

**Supplementary Information:**

The online version contains supplementary material available at 10.1186/s12873-021-00494-4.

## Introduction

Pediatric patients make up approximately 10% of emergency medical services (EMS) transports in the United States, representing approximately three million children each year [1]. These pediatric patients require unique skills and pose specific challenges for prehospital providers, including different treatment protocols, medication dosing calculations, and sizing of equipment.

Caring for critically ill or injured children creates additional stress and anxiety for providers, which may contribute to provider burnout, medical errors, and adverse patient safety events [2, 3]. Recent national provider surveys demonstrated a concern for maintenance of airway management, general assessment skills, and managing anxiety related to critical pediatric calls [4]. In addition, most pediatric calls require few advanced skills for the management of the patient, leading to a decline in skill mastery [5–7]. Lack of exposure to a pediatric patient population can be an obstacle to optimal care [8, 9], exacerbated by the need for age- and weight-based sizing and dosing decisions [10]. In simulations of prehospital pediatric encounters, this has been shown to contribute to errors such as incorrect medication dosing [11–13].

The 2007 Institute of Medicine’s consensus report, “Emergency Care for Children,” reported deficiencies in pediatric prehospital care, likely resulting from infrequent encounters as well as inadequate pediatric-focused continuing education [14]. The National Association of EMS Officials (NASEMSO) created an EMS Education Toolkit for Pediatrics, aimed “to improve evaluation and performance related to pediatric skills competency” based on the National EMS Educational Standards [15]. EMS educators also maintain that regular cognitive and psychomotor learning opportunities be provided to paramedics for pediatric encounters [16]. Despite growing efforts to improve prehospital care for children, current training requirements and pediatric encounter exposure for EMS systems may vary across states or jurisdictions [9, 17]. These requirements often include completion of a pediatric advanced skill course such as the American Heart Association’s Pediatric Advanced Life Support (PALS) or the American Academy of Pediatrics’ Pediatric Education for Prehospital Professionals (PEPP), in additional to maintenance of Continuing Education (CE) requirement hours. However, there remains variation in the frequency and type of psychomotor testing for prehospital pediatric skills, with pediatric-specific psychomotor skills testing being more common in EMS agencies that respond to a higher pediatric call volume [18].

The use of patient scenarios with high-fidelity simulation is shown to be of benefit in maintaining high-risk, infrequently-practiced pediatric patient care skills [19–21]. Among prehospital providers, there is a growing body of literature to support that simulation is a positive training method [22], and its use has been shown to improve performance among prehospital providers in pediatric mass casualty triage [23], pediatric airway management [24, 25], and pediatric seizure management [26]. Doughty et al. developed a simulation-based education program for prehospital providers in Texas that included high-fidelity scenarios focused on neonatal resuscitation, respiratory distress, seizures, and non-accidental trauma; posttest results showed significant improvement in medical knowledge over pretest scores, as well as high levels of learner satisfaction [27].

Given the challenges and anxiety related to providing care to critically ill and injured children in the prehospital arena, the relative paucity of pediatric encounters for prehospital providers, the limited required training for prehospital providers, and the potential for high-fidelity simulation as an effective education tool, this study used both quantitative and qualitative methods to assess the need for a high-fidelity simulation-based curriculum in one urban EMS system.

The objective of this study was to use a mixed-methods approach to describe the current experience of EMS practitioners caring for critically ill and injured children, and the potential role of a simulation-based curriculum to improve pediatric prehospital skills. The study used data from three sources: a retrospective review of local pediatric EMS encounters over one year, survey data of EMS providers regarding their comfort level with caring for pediatric patients, and qualitative data from focus groups with EMS providers. Together this information was used to assess the experience of EMS providers caring for pediatric patients, find potential gaps in knowledge or common uncertainties, and assess the potential role of a simulation-based curriculum to improve pediatric prehospital skills. This analysis can be used to guide educational leaders in the development of a simulation based curriculum for this agency as well as similar large, urban EMS agencies.

## Methods

The study took place from July 2018 to February 2019 in San Francisco, California. Approval for this study, including a waiver of informed consent for use of the retrospective dataset of pediatric encounters, was obtained from the Institutional Review Board of the University of California, San Francisco. Electronic informed consent was obtained from all study participants. Data were obtained from three sources in a single, urban EMS system: a retrospective review of local pediatric EMS encounters over one year; survey data of EMS practitioners’ comfort with pediatric skills using a 7-point Likert scale; and qualitative data from focus groups with EMS practitioners assessing their experiences with pediatric patients and their preferred training modalities.

### Local EMS system

The San Francisco Emergency Medical Services System serves the City and County of San Francisco, an area of 47 mile^2^ with a daytime population of approximately 1.3 million and a night time population of 800,000. The 9–1-1 Emergency Medical Services call volume (demand for ambulance service) is approximately 120,000 calls annually, of which approximately 4% are for patients under the age of 18 years. All 9–1-1 calls in San Francisco are answered and dispatched by a single entity, The Emergency Communications Center at the Department of Emergency Management. Three EMS organizations respond to 9–1-1 calls: San Francisco Fire Department (SFFD, 75% of call volume), King American Ambulance Company (King, 15% of call volume), and American Medical Response (AMR, 10% of call volume). In addition, there are 4 additional private ambulance companies that provide interfacility transport for adult and pediatric patients that are not tracked by the San Francisco EMS Agency. All 9–1-1 patients are transported to one of 13 receiving hospitals. Among these, there is one Level 1 Trauma Center that serves both adults and children, as well as two pediatric critical care centers.

### Review of local Pediatric EMS encounters

All medical 9–1-1 calls managed by SFFD are logged using an electronic database (ESO, Austin, TX, USA). This database does not include data from calls managed by AMR or King. The SFFD database is compiled from the electronic EMS charts completed by the prehospital care team. We retrospectively analyzed the patient characteristics of all SFFD prehospital encounters for patients 18 years and younger from January 1, 2018 to December 31, 2018. The following characteristics were obtained and summarized as percentage of total numbers: time and date of encounter, patient’s age, gender, race/ethnicity, chief complaint, the primary impression (primary complaint as specified by the prehospital provider), acuity of transport to receiving hospital (low, emergent, or critical), and patients’ level of distress (mild, moderate, severe). Race/ethnicity data were self-reported by patient or caregiver and documented by EMS provider into predefined categories within electronic EMS chart (ESO, Austin, TX, USA).

The most frequent primary complaints were calculated for all encounters and for the following age groups: 0 to 1 years of age, 2 to 5 years of age, 6 to 11 years of age, 12 to 15 years of age, and 16 to 18 years of age. Some primary impressions were independently assigned to broader categories by two investigators (KP, NG) to better reflect the most common type of encounter. Any discordance was discussed and agreed upon by all investigators. For example, “trauma” included all primary impressions involving injury, burn, or hematoma. “Neurology” included primary impressions of headache and altered mental status, but excluded seizures given the high prevalence of seizures among pediatric EMS encounters.

### Survey design and collection

An electronic survey was developed to obtain information on EMS practitioners’ comfort with caring for critically ill pediatric patients. Respondents were asked to use a seven-point Likert scale (ranking from “extremely uncomfortable” to “extremely comfortable”) to rate 34 aspects of pediatric care divided into six clinical domains based on EMS education standards [28]: respiratory, shock, cardiac arrest, care of the newborn, trauma, and other (“other” included skills related to seizure management, using length-based weight estimation, toxidromes, and managing concerned parents).

The survey was initially piloted on a group of 6 EMS practitioners, including leadership representatives from each of the three EMS providers in San Francisco, and revised to reflect common feedback. The survey was distributed to EMS practitioners electronically using a secure email link through a local EMS listserv comprising approximately 400 members, as well as through a San Francisco EMS social media group with approximately 458 members. An estimated 858 possible EMS providers were eligible to participate in survey, though an unknown number of providers belonged to both email listserv and social media group. Participants were eligible to complete the survey if they were an active EMS practitioner working in San Francisco. There is total of approximately 2400 registered EMS providers in San Francisco. The total active workforce is unknown; local EMS providers are not required to have an email address or belong to unified directory. The survey was live from August 1, 2018 to October 30, 2018.

### Qualitative focus groups design and analysis

We conducted focus groups with a convenience sample of prehospital providers at each of the three major EMS agencies in San Francisco: SFFD, King American, and AMR. Participants were approached in-person by investigators after change of shift. In order to reflect the mixed nature of EMS teams, each group included a mix both Emergency Medicine Technicians (EMTs) and Paramedics. Given participants were approached after change of shift, each group contained participants from a single agency. No other persons were present during the focus groups aside from the researchers and participants. The focus groups were planned to comprise a minimum of three and maximum of twelve participants and were facilitated by one or more authors trained in qualitative interview techniques (KP, JB, and NG). Each group lasted between 30 to 60 min. They were continued until no new themes emerged from interviews, thereby precluding a predetermined number of participants consistent with qualitative methods [29]. Sampling from each agency reflected the volume of calls at each agency (for example, approximately three-quarters of sessions took place with SFFD providers).

The focus group guide was developed by the authors with support from other medical educators with experience in qualitative methods, and in accordance to prior qualitative research guidelines [30]. The guide was piloted on six EMS practitioners and edited to reflect common feedback (Appendix 1). All focus groups started by asking participants to recall their last encounter of a critically ill pediatric patient. Participants were prompted to discuss the case in detail and elicit challenges in caring for pediatric patients. The second half of the interview focused on training and learning, including types of learning formats that are beneficial, and prior experience with simulation-based exercises.

All focus group sessions were recorded and transcribed (RevRecorder, San Francisco, CA, USA). A “constant comparison” analysis was conducted by three authors (KP, NG, JB), whereby transcripts were repeatedly read and an initial framework of key codes was developed and refined with successive readings. Mutually agreed-upon definitions and examples for each code were developed; codes were reviewed and revised with disagreements in coding resolved by team consensus. Final themes were determined using the principles of grounded theory that are common for qualitative analysis [29, 31]. After a final analysis, all themes were discussed with the present authors (KP, JB, CC, NG, AZ), as well as a larger interdisciplinary team, including EMS leadership, local EMS providers, emergency medicine providers, and medical educators with experience in qualitative analysis.

## Results

### Pediatric EMS encounters in 2018

There were a total of 2731 recorded pediatric prehospital encounters by SFFD during the 2018 calendar year (Table 1). 1374 (50.3%) patients were male. The majority of encounters were lower acuity (2291, 83.9%); 2.1% (*n* = 57) of encounters were “critical,” the highest acuity level. The most frequent primary impression among all age groups was trauma (976, 35,7%), followed by neurologic excluding seizures (260, 9.5%), seizure (219, 8.0%), respiratory (210, 7.7%) and fever (193, 7.1%) (Table 2a). Among the predefined age groups, trauma remained the top primary impression in each group. Zero to 1 years of age had a higher number of dermatologic primary complaints, while older age groups had more complaints related to psychiatric needs, or toxicology/drug or alcohol use (Table 2b).

**Table 1:** Total Pediatric (age ≤ 18 years) EMS Encounters in 2018 from Major Urban EMS Agency (*N* = 2731)

	N	%
**Age**		
0–1	465	17.0%
2–5	446	16.3%
6–11	428	15.7%
12–15	513	18.8%
16–18	879	32.2%
**Gender**		
Female	1225	44.9%
Male	1374	50.3%
Not Reported	132	4.8%
**Race/Ethnicity**		
White	606	22.2%
Black or African American	450	16.5%
Hispanic or Latino	380	13.9%
Asian	284	10.4%
Native Hawaiian or Other Pacific Islander	22	0.8%
American Indian or Alaska Native	3	0.1%
Unknown	986	36.1%
**Distress**		
Mild	1346	49.3%
Moderate	364	13.3%
None	555	20.3%
Severe	79	2.9%
Not Reported	387	14.2%
**Final Patient Acuity**		
Critical (Red)	57	2.1%
Emergent (Yellow)	375	13.7%
Lower Acuity (Green)	2291	83.9%
Dead Without Resuscitation Efforts (Black)	3	0.1%
Not Reported	5	0.2%
**Medical Trauma**		
Medical	1728	63.3%
Medical & Trauma	59	2.2%
Trauma	931	34.1%
Not Reported	13	0.5%
**Quarter**		
Q1	743	27.2%
Q2	671	24.6%
Q3	635	23.3%
Q4	682	25.0%

**Table 2:** Most Frequent Primary Impression for all pediatric age groups (a) and subgroups (b)

a. All Ages, ***n*** = 2731			
		n	%
**Trauma**		976	35.7%
Neurologic / AMS*		260	9.5%
**Seizure**		219	8.0%
**Respiratory**		210	7.7%
**Fever / Constitutional**^**#**^		193	7.1%
**b. By Age Group**			
**Age Group**	**Impression Classification**	**n**	**%**
**0–1 yrs**	Trauma	80	17.2%
	Respiratory	76	16.3%
(*n* = 465)	Seizure	70	15.1%
	Fever / Constitutional	66	14.2%
	Dermatologic	26	5.6%
**2–5 yrs**	Trauma	144	32.3%
	Seizure	66	14.8%
(*n* = 446)	Respiratory	59	13.2%
	Fever / Constitutional	31	6.7%
	Neurologic / AMS	28	6.3%
**6–11 yrs**	Trauma	230	53.7%
	Respiratory	40	9.4%
(*n* = 428)	Seizure	22	5.1%
	GI^♦^ / Abdominal pain	20	4.7%
	Fever / Constitutional	20	4.7%
	Neurologic / AMS	20	4.7%
**12–15 yrs**	Trauma	209	40.7%
	Neurologic / AMS	58	11.3%
(*n* = 513)	Psychiatric / Anxiety	47	9.2%
	Toxin/Drug/Alcohol	41	8.0%
	Fever / Constitutional	33	6.4%
**16–18 yrs**	Trauma	313	35.6%
	Neurologic / AMS	131	14.9%
(*n* = 879)	Toxin/ Drug / Alcohol	130	14.8%
	Psychiatric / Anxiety	72	8.2%
	GI / Abdominal	54	6.1%

“No complaint” or missing primary impression, *N* = 211 (7.73%)

* AMS = Altered Mental Status

^#^ Constitutional includes “malaise” and “fatigue”

^♦^ GI = Gastrointestinal

### Likert scale survey

At total of 142 respondents opened the survey link. Four respondents declined participation after the informed consent page, and two ended the survey before answering any questions, leaving a total of 136 total respondents. This represents approximately 15.9% of eligible participants (*N* = 858) and approximately 5.0% of all prehospital practitioners registered in San Francisco. Table 3 describes the characteristics of the respondents. The majority of respondents were paramedics (*N* = 110, 80.9%). The majority of respondents (56.6%, *N* = 77) estimated participating in zero to two pediatric encounters per month.

**Table 3:** Characteristics of EMS Providers Responding to Quantitative Survey of Comfort with Caring for Pediatric Patients (*N* = 136)

	N	%
**Highest Level of Training**		
Paramedic	110	80.9%
EMT	24	17.7%
Other	2	1.5%
**Years of Experience**		
< 2	19	14.0%
2 to 5	40	29.4%
6 to 10	35	25.7%
11 to 15	18	13.2%
> 15	24	17.7%
**Self-Estimated Pediatric Encounters per Month**		
0 to 2 / month	77	56.6%
3 to 5 /month	48	35.3%
6 to 10 / month	8	5.9%
11 to 20 / month	3	2.2%
> 20 /month	0	0%

**Fig. 1:**
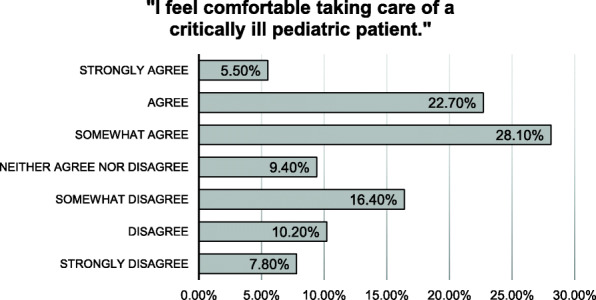
Likert Scale of EMS Practitioners’ Overall Comfort (*N* = 134)

Figure 1 demonstrates the Likert Scale responses for 34 queried skills. 34.4% of all respondents either somewhat disagree (16.4%), disagree (10.2%), or strongly disagree (7.8%) with the statement: “I feel comfortable taking care of a critically ill pediatric patient.” The majority of respondents felt some level of comfort with all queried skills (Appendix 2). Skills with highest level of respondents feeling “extremely comfortable” include: administering oxygen (70.3%), administering rescue breaths with a bag-mask-valve mask (46.5%), placing an airway adjunct (37.0%), performing chest compressions (35.5%), immobilizing the cervical spine (35.5%), controlling hemorrhage (35.5%), and using a length-based tape (41.2%). Skills with the highest cumulative level of discomfort include: placing an advanced airway (33.0%), placing an IV (29.1%), managing cardiac arrest (37.1%), delivering a newborn (32.2%), recognizing common toxidromes (35.8%), calculating the correct dose for administration of common resuscitation medications/fluids (33.4%), recognizing signs of increased intracranial pressure (30.6%), and management of a near drowning pediatric patient (29.8%).

### Qualitative focus groups

A total of 47 EMS practitioners participated in the qualitative focus groups: 26 from SFFD, 9 from AMR and 12 from King American. Each focus group contained both EMTs and Paramedics, with a minimum of 3 participants, a maximum of 9, and mean of 4.5. Three major themes emerged: experience with pediatric patients, procedural skills, and training and education (Table 4).

**Table 4:** Major Themes from Qualitative Focus Groups with 47 EMS Providers and Example Quotes

Domain and Themes	Example Quote
**I. Experience**	
1. Low number / lack of experience	*We definitely don’t run a lot of pediatric calls [...] they’re very low frequency calls so you do start to feel a little bit rusty [...] there’s that lack of contacts with pediatric patients […] I definitely can see how it becomes one of those things that’s like...Oh my gosh, this is a pediatric call in the city!*
2. Pediatric patients are fundamentally different / more stressful	*Even with a baby with totally stable vitals there does seem to be an air of anxiety among the responders*
3. Managing scene / more chaotic scene	*When you have them it’s a little bit chaotic and rowdy there especially when you don’t necessarily know the others that are there; you never even worked with them and there could be some communication issues as far as who’s doing what, too many cooks in the kitchen.*
4. Tendency to “grab and go”	*They just want to grab the baby and throw him in the ambulance and just tell us to go. You know, there’s so many things we’ve got to do beforehand - get the parents ready, check out the baby, do all our assessment. But they just want to grab him and just be like, “All right you guys ready to go?”*
**II. Procedural Skills**	
1. Challenge of medication dosing	*Not just what is the dose for this drug, but how are you going to draw it up, how is it formulated, what is the volume you draw up, what concentration, something a lot of people don’t really think about until it comes time to do it and they’re “oh, I totally forgot how much percent do I give?”*
2. Communication with parents, caregivers, and patient	*One of the more challenging things, or the added challenges, with kids is not only you have the patient, but you have at least two or three patients, which are the parents, the family. Which is often overwhelming, especially if they are emotional or trying not to give the child up. I feel like the environment is just very different. You often have more than one person to take care of. And I feel like if you’re not used to that or if people don’t know that environment, it might make it more challenging*
3. Airway management	*Anything airway, we don’t have as many tools to manage a baby’s airways like we do for an adult. With adults, I have a lot of things I can do for somebody; as for a kid? Not so many. That would be my biggest thing is give me more tools to manage a bad airway*
**III. Training and Education**	
1. Need for repetition, reinforcement, and feedback	*In order to keep those skills proficient, there has to be a lot of frequent ongoing training.[…] That’s where I think a lot of the providers are deficient in their skills. They’re not as confident because there are not enough training hours to support this large volume of skills that you have to maintain.*
2. Preference for hands-on and small group setting	*You know right off the bat, I think having that hands on, holding something in your hand experience, really, really cements how things are supposed to go.*
3. Desire for more interaction with physicians and hospital setting	*What we need more frequently is building that relationship between pre-hospital, medics, firefighters, everyone out there. Bridging that gap between the actual hospital providers and the emergency room. Knowing what they require of us, what information they would want in certain situations.*
4. Receptive to high fidelity simulation	*For me it would definitely be a strong preference for simulation. Simulation, simulation, simulation! Muscle memory of just going through calls. Doing a PALS class once a year is just not…it’s so woefully not enough. Just having the monitor, having the mannequin set up, calculating drug doses, running codes is important to practice.*
5. Suspicion for more training / desire for on-job training	*I would put the emphasis on in-service training because most of us are family people, we have kids. […] So I’ve got a kid, school, work. If it’s outside my work [...] I’m probably not going to go to a training. I’m going to look and say, okay I’ve got this assignment, or I got this [...] That can try to get me on a day off, but I’m not going to do it*

### Experience with pediatric patients

Participants felt a general lack of experience with pediatric patients and a low number of pediatric response calls. As one participant explained:

*“I mean we have all these tools, but we don't use them in frequent skills. So if you don't use it, you're going to lose it eventually “*

EMS providers also found pediatric patients fundamentally more stressful:

*“You show up and you're like no one said it was a kid. […] There's just a ton of extra stuff you have to do with kids even though the situation itself might be minuscule...“*

Many remarks also involved managing a more chaotic scene, which often includes more family members as well as crew members.

*“…it's hectic enough for us having to run the call and decide what we're going to do with this sick child without having to try and manage a whole team of people you don't know and don't really know if they're on the same page with you or not.*”

Given the lack of volume and more chaotic scene, providers often felt there was a tendency to “grab and go.” As one explained:

*“So often these calls you show up on scene and it's just engine, medic or EMT running out of the house with the kid in their hands with nothing done…”*

### Procedural skills

The most common theme for procedural skills related to the dosing of medication for pediatric patients. One provider commented:

*“Keeping up on your skills and remembering doses, dosages and medications and that. Just keep that in the back of your mind. Adult cases we do all the time but not pediatrics.”*

Providers also expressed the challenge of effectively communicating with parents, caregivers and the patient:

*“I often find that dealing with parents and their emotions around the entire experience is one of the hardest things, and I don't know about any of you but I don't remember ever even discussing that as part of my training.”*

Other challenging skills that were common for providers included: managing the pediatric airway, inserting peripheral IVs, intraosseous access, using the Broselow length-based tape, knowing local pediatric based protocols, and debriefing after stressful encounters.

### Training and Education

Participants expressed several concepts under the training and education domain. First, providers conveyed a need for repetition, reinforcement and feedback for pediatric training:

*“If there isn’t that frequent training or that open relationship with whatever agency you’re dealing with, then those skills are going to be perishable. They’re super perishable.”*

Participants also expressed a preference for hands-on training, as one paramedic explained:

*“For me it's like playing a game, playing sports, you kind of practice how you play with your teammates, then you have the big show now let's see it in practice.”*

EMS providers also articulated a desire for more interaction with physicians and the hospital setting.

*“I think there's a lot of value having a physician get involved and actually present the material and do ongoing CE [continuing education] […] because the response is different when you present it versus another department trainer.”*

When specifically asked, EMS providers were receptive to high-fidelity stimulation:

*“I think if you have a good sim lab and you can change skin color and pupils and respirations and all this stuff […] so you're using your eyes, your hands, your ears, and everything prompted you have […] a good sim lab is a way to hone your assessment skills.”*

Despite this desire for more training and openness to high-fidelity stimulation, participants did express a desire for additional training to be conducted during work hours; there were clear concerns about the challenges of attending additional training during time off.

## Discussion

This mixed-methods study offers a comprehensive assessment of the experience of EMS providers caring for pediatric patients using three data sources: a retrospective review of local pediatric EMS encounters over one year, survey data of EMS providers regarding their experience with caring for pediatric patients, and qualitative data from focus groups with EMS providers.

All three data sources reinforced low numbers of critical pediatric encounters. This lack of real-world call volume supports the potential value for high-fidelity simulation as a training tool, consistent with a benefit in maintaining high-risk, infrequently-practiced patient care skills [19–21].

Providers expressed several skills that could be the focus of a simulation-based curriculum. These skills were largely consistent across both the qualitative analysis as well as surveyed domains, including placement of an advanced airway, managing pediatric cardiac arrest, and determining correct doses of medications. Prior studies have already examined the use of simulation to improve some of these skills. For example, Stopyra et al. used high fidelity simulation to teach both rapid sequence intubation (RSI) [24] and needle cricothyrotomy [25] to prehospital providers. Simulation has also been used to demonstrate effectiveness of using a laryngeal mask airway, Pedi-King tube placement, and endotracheal intubation among prehospital providers [32–35]. A prospective study of simulation-based pediatric trauma resuscitation performed at three community emergency departments found that provider comfort improved with infant airway, infant IV access, and Broselow tape use, among other skills [36].

While over half of surveyed EMS providers noted some level of comfort with taking care of critically ill pediatric patients (56.3%, Fig. 1), focus groups revealed elements of discomfort with many aspects of caring for pediatric patients. This disparity could be explained by the fact that while individual perceived comfort for certain skills may be adequate, deeper reflection upon specific, real-world experiences caring for a critically ill child reveals unrecognized distress. This may be particularly true for skills or aspects of care that are not routinely part of recognized educational standards. For example, one skill that demonstrated disparate findings between the survey and focus groups was effective communication with parents and caregivers. Among surveyed providers, the majority (73.4%) expressed feeling comfortable with the skill, “managing the concerns of the parents of sick child” (Appendix 2f). However, one of the primary themes derived from focus groups was that communication with parents, and managing an increased number of providers and caregivers on scene, were frequent challenges. This inconsistency represents the limited scope of the surveyed question as well as the inability of closed-ended survey questions to provide nuanced responses. Prior qualitative research has further elucidated the experiences of prehospital providers and their perceived barriers to effective patient- and family- centered care [37]. Simulation-based exercises may offer a means to improve communication and scene-management skills, allowing providers to have direct feedback from parent actors.

Despite growing efforts to improve EMS pediatric-specific education, including simulated exercises and psychomotor skill testing, there remains no national standard on the initial certification and recertification of pediatric-specific EMS education [18, 38]. This study reveals that EMS practitioners yearn for better, and more frequent, learning modalities. These could include high-fidelity simulation and more interaction with physicians and other health providers. Prehospital providers in this study recognize the importance of maintaining pediatric skills through hands-on exercises and are motivated to provide the best prehospital care for critically ill and injured children. Further research should elucidate the effectiveness of specific simulated scenarios on skill improvement and retention, as well the ideal context and frequency to incorporate simulation-based learning into a prehospital curriculum.

### Limitations

The present study examined a single, urban city in California, which may limit the generalizability of these results. San Francisco, in particular, may have unique demographic characteristics and an unusually low volume of pediatric calls compared to other urban EMS agencies. Similarly, the specific scope of practice of EMS providers has regional variation that may exclude certain procedures or skills in the present study. The review of EMS pediatrics encounters is subject to common limitations of retrospective chart reviews, including human errors in entering accurate information and the omission of data. The electronic survey of EMS providers may be subject to selection bias. The low response rate may also be impacted by the means of electronic distribution and lack of central directory for active EMS members. The focus groups are also subject to selection bias of a convenience sample of EMS providers that may not be representative of all EMS practitioners (this favors those more willing to participate and engage in the discussion). We attempted to limit this bias by using a selection of providers at each of the three major EMS organizations at different times, with a variety of training and experience. In addition, we used “triangulation,” or the use of multiple data sources, in our investigation, to offer a deeper understanding [39].

## Conclusion

Prehospital encounters with the critically ill pediatric patient are rare events. In this mixed methods study of urban EMS practitioners, discomfort with caring for critically ill children was found to be a major theme, despite the majority of providers reporting comfort on surveyed skills. This dissonance may be due to the inherent limitations of interpreting survey data and requires further investigation. Prehospital providers are receptive to training with high-fidelity simulation, which may be an effective means to improve comfort levels and proficiency of pediatric skills. Any future prehospital simulation-based curriculum should focus on those specific skills which show the least comfort, the lowest retention, and the most critical consequences for patients and practitioners.

## Supplementary Information

**Additional file 1.**

## Data Availability

The datasets generated during the current study are not publicly available to ensure anonymity of study subjects. While data is deidentified, some identifiers may be deduced in this small, specific study population. Datasets are available from the corresponding author upon reasonable request.
