# Supplementary material for: Development of a simulation-based curriculum for Pediatric prehospital skills: a mixed-methods needs assessment
Source: BMC Emerg Med. 2021 Sep 25;21:107. doi: 10.1186/s12873-021-00494-4 (PMC8465767; doi:10.1186/s12873-021-00494-4)

**Title: Development of a Simulation-Based Curriculum for Pediatric Prehospital Skills: A Mixed-Methods Needs Assessment**

**Author List:**
Kevin A. Padrez, MD [1]
John Brown, MD [1, 2]
Andy Zanoff, MICP [3]
Carol C. Chen, MD, MPH [1]
Nicolaus Glomb, MD, MPH [1]

**Author Affiliations:**
[1] University of California, San Francisco, Department of Emergency Medicine
[2] San Francisco EMS Agency
[3] San Francisco Fire Department

**Corresponding Author:** Kevin A. Padrez, kevin.padrez@ucsf.edu

**Keywords:** Simulation, pediatrics, education

**Word Count:** 3528

<u>**Declarations**</u>
**Ethics approval and consent to participate:** Ethical approval for the current study was obtained from Institutional Review Board of The University of California, San Francisco. Informed consent was obtained from study participants and informed consent waiver for the prehospital call dataset was obtained from Institutional Review Board of The University of California, San Francisco.

**Consent for publication:** Not applicable

**Availability of data and materials:** The datasets generated during the current study are not publicly available to ensure anonymity of study subjects. While data is deidentified, some identifiers may be deduced in this small, specific study population. Datasets are available from the corresponding author upon reasonable request.

**Competing interests:** None

**Funding:** None

**Authors' contributions:** KP, JB, and NG wrote main manuscript text. KP, JB, AZ, CC and NG assisted in study design and implementation, data analysis, preparation of all tables and figures, and review of the manuscript.

**Acknowledgements:** San Francisco Fire Department, King American Ambulance Company, American Medical Response. Thanks to Esther H. Chen for her input on focus group guide and qualitative methodology

## Appendix 1: EMS Focus Group Leader Guide

I. Introductions

1. Introduce yourself. Ask group to introduce themselves, including company, training, years of experience.

II. Explanation

*This is a focus group about caring for pediatric patients in the prehospital setting. We hope this interview will guide our educational team as we design simulation-based trainings to best serve you and your colleagues in the future. We are going to ask some questions about your experience, skills, emotions and uncertainties around caring for pediatric patients. It is important to know that, although we are recording, all of your replies are confidential and will be kept anonymous in our final investigation. After the session we will analyze the conversations to find common themes and key messages. We are also collecting this information for research purposes with the intention to publish the findings of our needs assessment, including these focus group discussions. Your participation is completely voluntary. Again, no information you give us will be shared with anyone else, and will not lead to any punitive or disciplinary actions. You can decline to answer any questions or stop the interview at any time.*

III. Last Encounter / Primer

1. Think back to the last time you were called for a critical pediatric patient. What was the call for? Describe the encounter. What interventions did you make? What went well with the encounter? What was most challenging about the encounter?

IV. Difficulties

1. What are the challenges in caring for pediatric patients?
2. What do you find most difficult about the assessment of pediatric patients?
3. What procedures or skills do you find most difficult in pediatric patients?
4. *For paramedics: In your experience of supervising or training other EMS providers, what do you think are the common pitfalls that young EMS providers often make when caring for pediatric patients?*
5. How have you dealt with these challenges?

V. Training

1. When thinking about how you learn best, what kind of teaching formats do you prefer? ( if needed, give examples: e.g. small group sessions, case scenarios, high fidelity simulation, lecture, etc.). Why?
2. What experiences have you had with simulation exercises? What specific skills do you think simulation training might be good for? Why or why not?
3. What pediatric clinical scenarios do you think would be most beneficial for you and your company to practice using high fidelity simulation? Why?

4. Up to now, how were you taught pediatric procedures? What is most challenging about learning pediatric procedures? Which specific procedures would be most beneficial for your clinical practice?

VI. Final

1. Is there anything else that we did not ask about that you would like to add about caring for pediatric patients in the prehospital setting?

Suggestions for leading focus groups:
- Summarize what you think you have heard, and ask if the group agrees
- Phrase the same question in a different way if there is no response
- Ask if anyone else has any comments on that question
- Ask all relevant follow-up questions
- Look around the room, and make brief eye contact, especially with those who may not have spoken

**Appendix 2: Likert Scale of EMS Providers' Comfort with 34 Pediatric Skills Under Six Domains (N = 134)**

**Key**

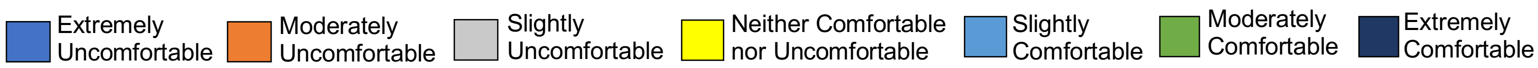


## a. Respiratory Skills

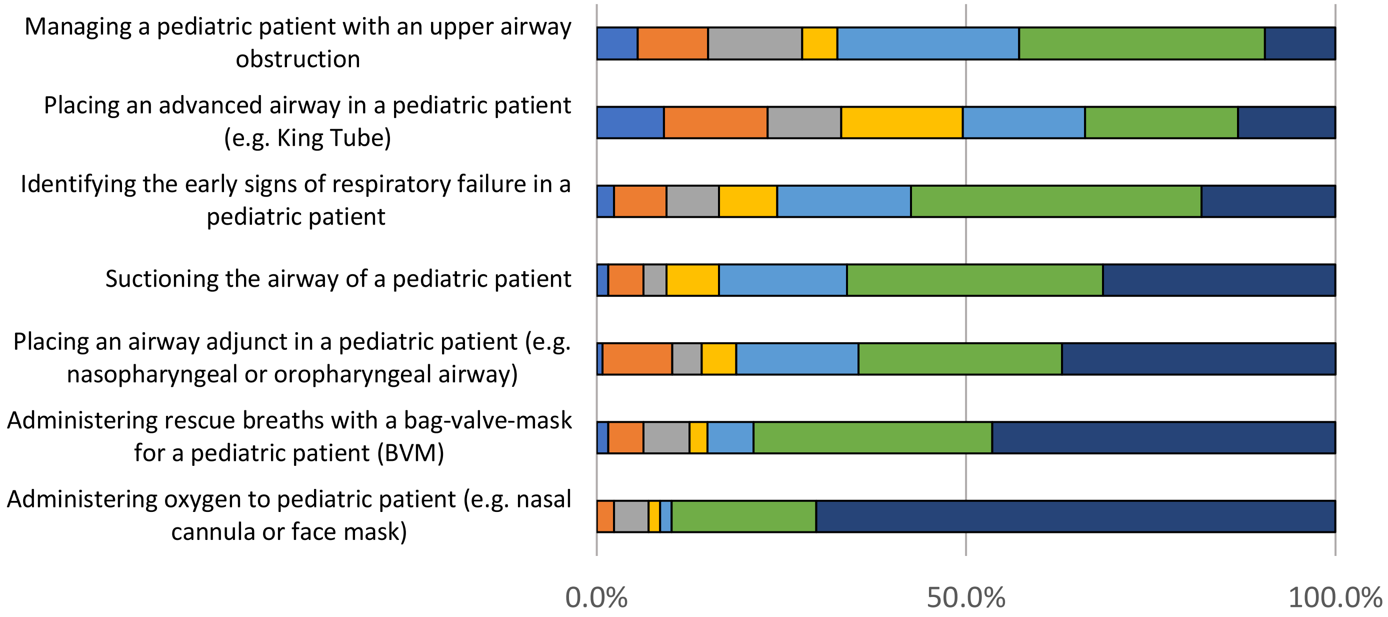


## b. Shock Skills

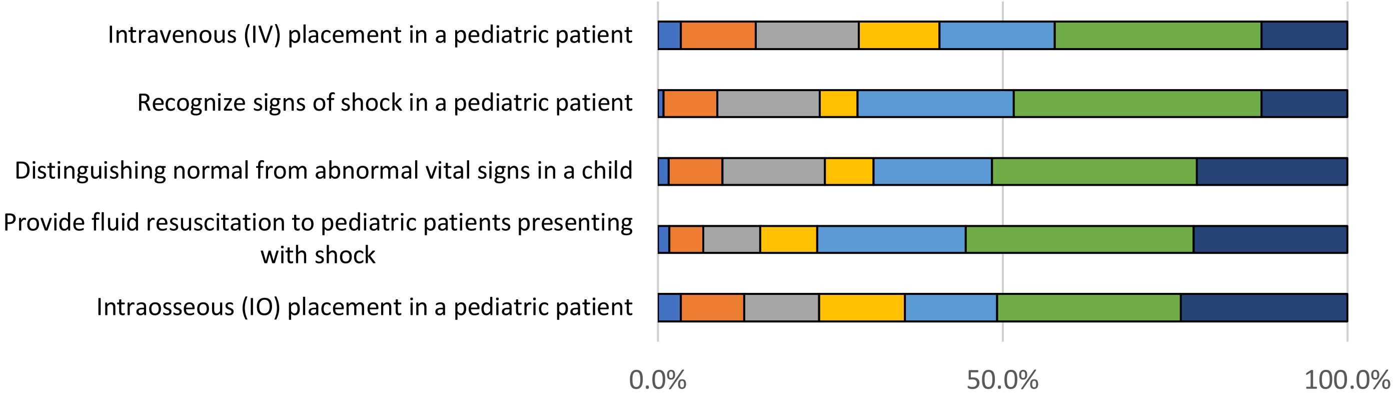

## c. Cardiac Arrest

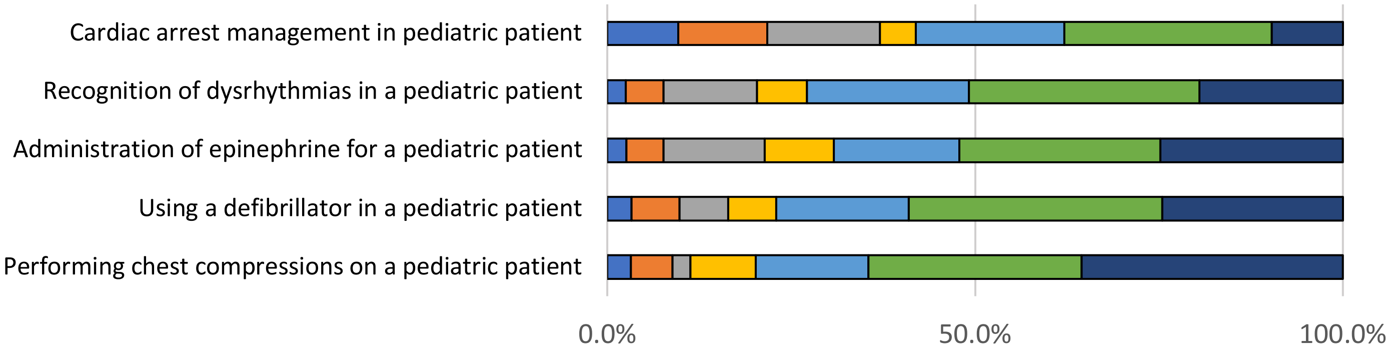


## d. Newborn Care

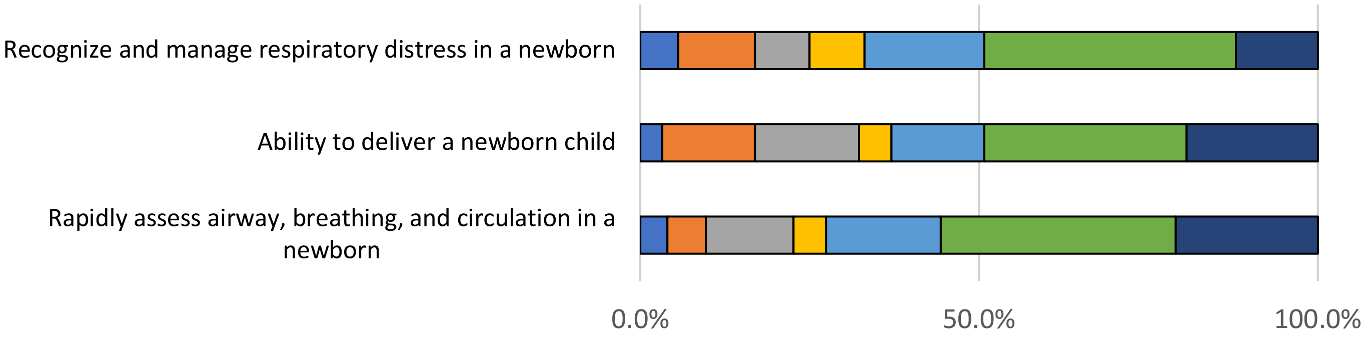


## e. Trauma Skills

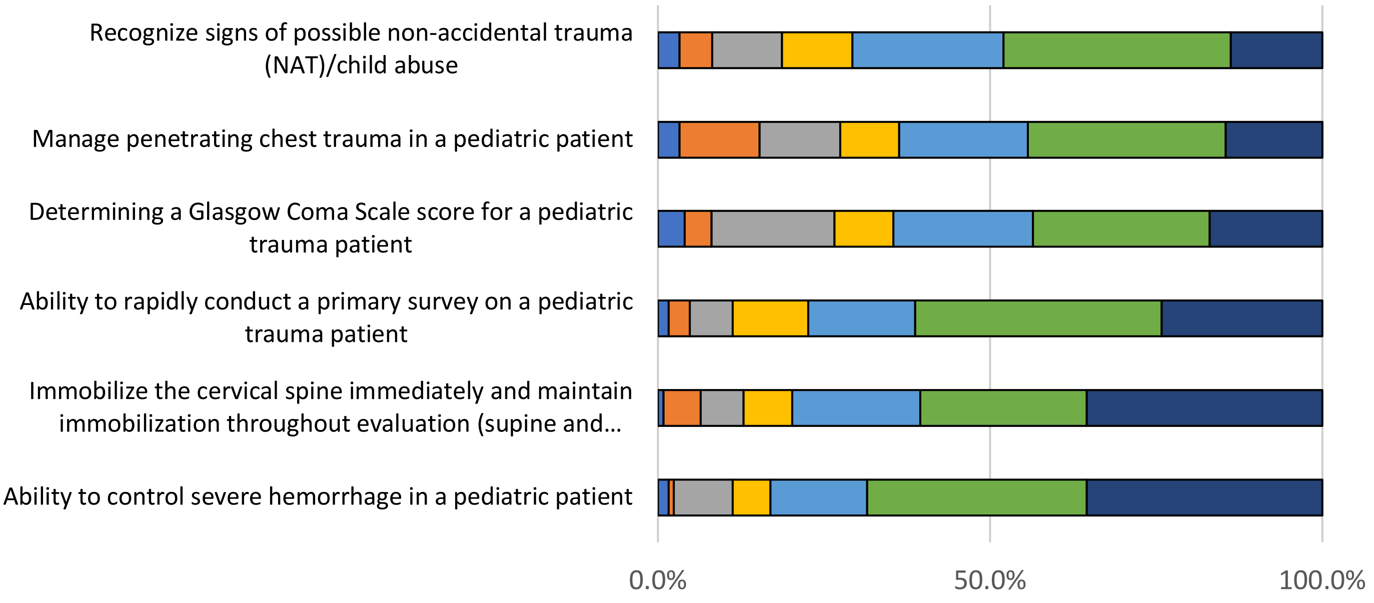

## f. Other Skills

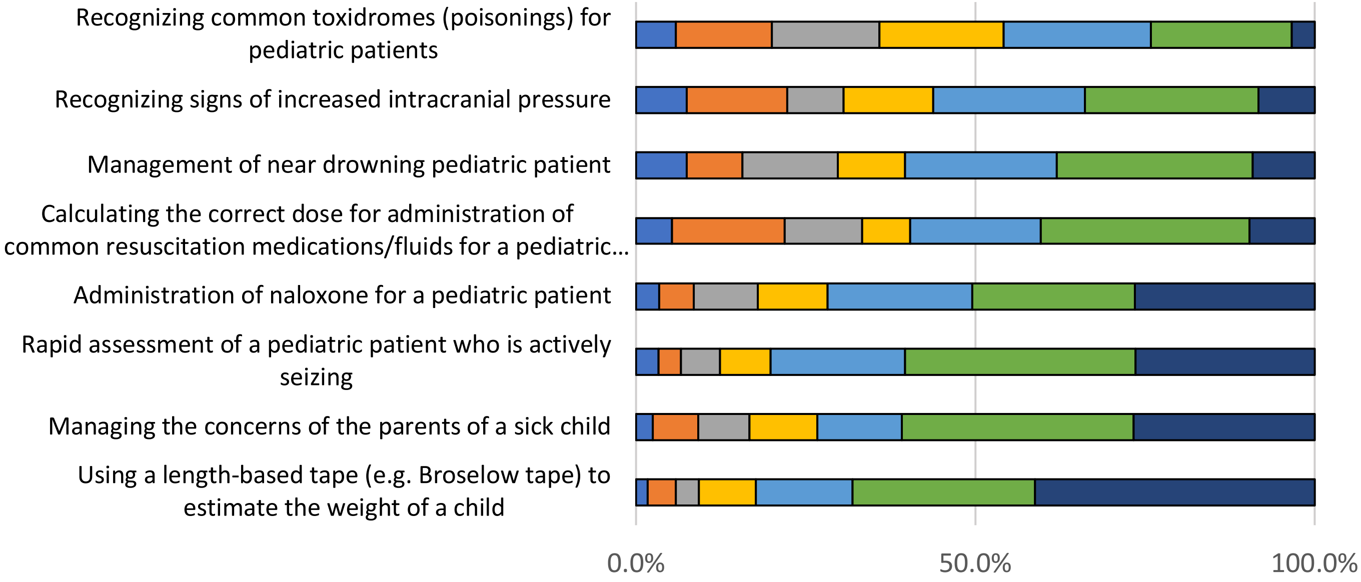

Supplement: Supplementary file 1 — Additional file 1. [file 12873_2021_494_MOESM1_ESM.pdf]
